# In vitro evaluation of two novel *Escherichia* bacteriophages against multiple drug resistant avian pathogenic *Escherichia coli*

**DOI:** 10.1186/s12879-024-09402-0

**Published:** 2024-05-16

**Authors:** Mobina Karami, Ali Goudarztalejerdi, Abdolmajid Mohammadzadeh, Enayat Berizi

**Affiliations:** 1 Department of Pathobiology, Faculty of Veterinary Medicine, Bu-Ali Sina University, Hamedan, 6517658978 Iran; 2 Department of Food Hygiene and Quality Control, School of Nutrition and Food Sciences, Shiraz University of Medical Sciences, Shiraz, Iran

**Keywords:** Avian pathogenic *Escherichia* coli (APEC), Antibiotic, Bacteriophage, Broilers, Colibacillosis, Multidrug resistant (MDR)

## Abstract

**Background:**

In recent years, there has been a growing interest in phage therapy as an effective therapeutic tool against colibacillosis caused by avian pathogenic *Escherichia coli* (APEC) which resulted from the increasing number of multidrug resistant (MDR) APEC strains.

**Methods:**

In the present study, we reported the characterization of a new lytic bacteriophage (*Escherichia* phage AG- MK-2022. Basu) isolated from poultry slaughterhouse wastewater. In addition, the in vitro bacteriolytic activity of the newly isolated phage (*Escherichia* phage AG- MK-2022. Basu) and the *Escherichia* phage VaT-2019a isolate PE17 (GenBank: MK353636.1) were assessed against MDR- APEC strains (*n =* 100) isolated from broiler chickens with clinical signs of colibacillosis.

**Results:**

*Escherichia *phage AG- MK-2022. Basu belongs to the *Myoviridae* family and exhibits a broad host range. Furthermore, the phage showed stability under a wide range of temperatures, pH values and different concentrations of NaCl. Genome analysis of the *Escherichia* phage AG- MK-2022. Basu revealed that the phage possesses no antibiotic resistance genes (ARGs), mobile genetic elements (MGEs), and any *E*. *coli* virulence associated genes. In vitro bacterial challenge tests demonstrated that two phages, the *Escherichia* phage VaT-2019a isolate PE17 and the *Escherichia* phage AG- MK-2022. Basu exhibited high bactericidal activity against APEC strains and lysed 95% of the tested APEC strains.

**Conclusions:**

The current study findings indicate that both phages could be suggested as safe biocontrol agents and alternatives to antibiotics for controlling MDR-APEC strains isolated from broilers.

**Supplementary Information:**

The online version contains supplementary material available at 10.1186/s12879-024-09402-0.

## Background

Avian collibasilosis is one of the most important causes of morbidity, mortality, and considerable economic loss in the poultry industry worldwide [1, 2]. This complex syndrome is characterized by colisepticemia, coligranuloma, pleuropneumonia, salpingitis, omphalitis, pericarditis, peritonitis, airsacculitis, perihepatitis, and swollen head syndrome in chickens [2], and osteomyelitis complex in turkeys [3]. Avian pathogenic *Escherichia coli* (APEC) is a subdivision of extraintestinal pathogenic *E. coli* (ExPEC), known as the main etiological agent of avian collibasiosis [2, 4]. The APEC strains have also been reported as potential zoonotic pathogens [5, 6].

Over the past decades, antibiotics have traditionally been used for the treatment and control of bacterial infections; however, the long-term and excessive use of antibiotics has led to an increase in antimicrobial resistance (AMR), and subsequently, bacteria show high multidrug resistance (MDR) properties, which poses a serious concern for animal, human and public health [7–9]. To combat these emerging MDR bacteria, novel strategies and alternative treatments, such as phage therapy (using bacteriophages to target and kill specific bacteria), are urgently needed [10–12]. Recently, the biocontrol of bacterial pathogens using bacteriophages (short phages) has attracted growing interest as an alternative to conventional antibiotic therapies, particularly against MDR bacterial pathogens [13–16]. There are 10^31^ bacteriophage particles in the biosphere, which are the most abundant on Earth [17, 18]. Phage therapy has several potential advantages compared to traditional antibiotic therapy. Some of these advantages include the following circumstances: (i) highly specific targeted; phages can be engineered to target specific bacteria, which means that they can be more effective in eliminating the specific pathogen causing an infection while leaving other bacteria in the body intact; (ii) a lower risk of resistance; bacteria can develop resistance to antibiotics over time, however because phages target specific bacteria, the risk of resistance development is lower; (iii) fewer side effects can occur; antibiotics can sometimes cause side effects such as stomach upset, diarrhea, or allergic reactions, however, phages are typically less likely to cause side effects, and (iv) availability; phages are naturally occurring viruses that can be found in soil, water, and other environments, which means that they are widely available and relatively easy to isolate [11, 19–21].

In recent years, avian collibasilosis prevalence, molecular characterization and MDR properties of APEC strains have been reported in Iran [7, 22–24], and considering this problem, applying new effective treatments for MDR- APEC strains is necessary. Therefore, the aims of this study were to (1) isolate and check the stability and characterize the physical and genetic properties of a new *E. coli*-specific phage (*Escherichia* phage AG- MK-2022. Basu), (2) determination of the phage host range and efficiency of plating of newly isolated *Escherichia* phage, and (3) evaluation of the bacteriolytic potential of the newly isolated phage (*Escherichia* phage AG- MK-2022. Basu) and the *Escherichia* phage VaT-2019a isolate PE17 (GenBank: MK353636.1), which was obtained from a recently published study [25], against MDR APEC strains, in vitro.

## Methods

### Bacterial strains

In this study, seven ATCC bacteria prepared from the strain collection of the Faculty of Veterinary Medicine (Bu-Ali Sina University, Hamedan, Iran) were used as reference strains (Table 1). In addition, a total of 100 APEC isolates obtained from previous research [24] were used for the studies. All the strains were revived by subculture in Tryptic Soy Broth (TSB; Merck, Germany) at 37 °C for 24 to 48 h separately.

**Table 1:** Bacterial strains used for the host range determination of the *Escherichia* phage AG- MK-2022. Basu

Bacterial strain	Infectivity of phage
*Escherichia coli* ATCC^1^ 25,922	+
*Escherichia coli* serotype O157: H7 ATCC 43,895	+
*Staphylococcus aureus* ATCC 25,923	-
*Salmonella enterica* subsp. *enterica serovar Typhimurium* ATCC 14,028	+
*Proteus mirabilis* ATCC 43,071	
*Pseudomonas aeruginosa* ATCC 27,253	+
*Enterococcus faecalis* ATCC 29,212	-

“+” indicates indicate positive sensitivity to phage lysis, and “−” indicates that did not form any plaque on the strain. ^1^ATCC: American Type Culture Collection

### Bacteriophage isolation, purification, and propagation

In this study, two bacteriophages, *Escherichia* phage VaT-2019a isolate PE17 and *Escherichia* phage AG- MK-2022. Basu, were used. *Escherichia* phage VaT-2019a isolate PE17 (GenBank: MK353636.1) was obtained from a recently published study [25]. The second phage, *Escherichia* phage AG- MK-2022. Basu was isolated and purified from sewage water collected from a poultry slaughterhouse in Hamedan, Iran, using a modified method as described previously [26]. Briefly, the sewage water samples collected from different parts of the poultry slaughterhouse were held at 4 °C for 24 h to allow large particles to sediment. Then, 100 ml of each sample was centrifuged (6000 rpm, 15 min, 4 °C), and the supernatant was passed through a 0.22 μm syringe filter (FilterBio® Sterile syringe filters, Nantong FilterBio Membrane Co, China). Subsequently, 100 µl of each filtered sample was mixed with 3 ml of *E. coli* ATCC25922 overnight culture supplemented with 10 mM MgSO4 and incubated at 37 °C at 160 rpm for approximately 5 h. Finally, the mixture was centrifuged at 6000 rpm for 10 min to remove bacterial cells, and the supernatant was filtered again. The filtered suspension was tested for the presence of bacteriophage using a double layer agar assay (DLA) as described previously [27]. The isolated phage was purified by picking a single plaque (lack of bacterial growth) from the plate, transferring it to TSB containing host bacteria, incubating it at 37 °C for 24 h, and then centrifuging (600 rpm, 10 min). The supernatant was collected and used as a source of isolated phage. The procedure was repeated three times to obtain pure phage cultures, and the purified phage was stored at 4 °C until use [28].

### Bacteriophage titer measurement

Titers of phage suspensions were then measured using the double-layer agar (DLA) technique as was previously described [27, 29]. Briefly, the phage suspensions were diluted (10^− 1^, 10^− 9^) using SM buffer (8 mM MgSO4⋅7H2O, 50 mM Tris–HCl, 100 mM NaCl) and 100 µl of each phage dilution plus 200 µl of host bacteria mixed with 3 ml melted 0/5% brain heart infusion (BHI) agar (Merck Millipore, Germany). The mixture was then poured onto solidified plates containing 1/5% BHI agar medium as a surface layer and incubated at 37 °C for 24 h. The number of lysed plaques was counted, and the result are reported as plaque forming units per milliliter (PFU/ml).

### Electron microscopy

Transmission electron micrographs were obtained according to a previously described procedure [30]. Briefly, a highly concentrated phage suspension (10^9^ PFU/mL) was centrifuged at 30,000 ×g for 3 h at 4 °C, the supernatant was poured off, the pellet was resuspended in 0.1 M ammonium acetate solution, and then the suspension was filtered (0.22 mm). Transmission electron microscopy analysis of the samples was performed by Transmission Electron Microscope (Zeiss, Germany) in the Laboratory of Electron Microscopy, Pasteur Institute, Tehran, Iran.

### Phage host range determination and efficiency of plating

The host range of the phage was determined by the spot method [31] using the bacteria listed in Table 1. Overnight cultures (100 µl) of each tested strain were inoculated on the surface of BHI agar plates, the plates were allowed to dry at room temperature, and then 10 µl of phage (10^9^ PFU/ml) was spotted in triplicate onto the surface of the plates. The plates were incubated at 37 °C and monitored for clearance zones. The appearance of clear single plaques was considered evidence of susceptibility to the phage. The efficiency of plating (EOP) is a measurement of the bactericidal efficiency of the bacteriophage on a given bacterial cell line compared to the host bacteria [31]. The EOP was determined for positive bacteria in the spot test using the DLA method [27]. The experiments were performed thrice for each strain. The EOP was calculated based on (average PFU on target bacteria/average PFU on host bacteria) [31]. The results were classified based on EOP values as high, moderate, low, or insufficient. (EOP ≥ 0.5, high efficiency; 0.1 ≤ EOP < 0.5, moderate efficiency; 0.001 < EOP).

### Phage stability

The stability of phage under different conditions, including temperature, pH, and NaCl, was evaluated using previously described methods [32, 33], with few modifications. To test the temperature stability of *Escherichia* phage AG- MK-2022. Basu. 10 µl of phage suspension (10^9^ PFU/mL) was incubated at temperatures ranging from 4 °C to 80 °C for 1 h, followed by determining phage titer and viability using the DLA method. The phage stability at different pH values was evaluated by mixing 10 µl of concentrated phage suspension (10^9^ PFU/mL) with 990 µl of SM buffer and adjusting at various pH values ranging from 1 to 14. The mixture was then incubated at 37 °C for 1 h, and then the phage titer was determined using the DLA method. The stability of the phage was tested against various concentrations of NaCl by incubating phage (10^9^ PFU/mL) with varying ratios of NaCl (1 to 11%) at 37 °C for 60 min, and the phage titer was assayed using the DLA method.

### Phage adsorption assay

To determine Phage adsorption assay of *Escherichia* phage AG- MK-2022. Basu, 1 ml of fresh host bacterial culture of *E. coli* ATCC 25,922 (10 ^9^ CFU/ml) was mixed with 10 µl of phage suspension (10^8^ PFU/ml) to reach a multiplicity of infection (MOI) = 0.1 and, then pre-warmed fresh TSB was added to mixture. The phage-host mixture incubated with shaking at 160 rpm and 37 °C. After 0, 5, and 10 min, 100 µl of suspension were collected, and added in 900 µl SM buffer, centrifuged at 6,000 g for 1 min and supernatants were filtered by using 0.2 μm membrane filter. The filtered liquid was cultured using the DLA method, and the titer of the nonadsorbed phages was calculated. The percentage of nonadsorbed phages at every given time was determined by dividing the phage titer at 5 and 10 min by the phage titer at time zero [34, 35].

### One-step growth curve

A one-step growth experiment was performed as described previously [32], with some modifications. In brief, a 1 ml log phase of the *E. coli* ATCC 25,922 (1.5 × 10^8^ CFU/mL) and, then a 1ml *Escherichia* phage AG- MK-2022. Basu suspension (10^6^ PFU/mL) were added to the host at an MOI = 0.1 and to allow phage adsorption to bacterial cells for 5 min at room temperature. The phage-bacterial mixture was centrifuged at 8000 × g for 5 min and then the supernatant was discarded. After three washes, the pellet was resuspended in 10 ml of pre-warmed fresh TSB and incubated with shaking at 180 rpm and 37 °C. Subsequently, 100 µl of the mixture were collected every 10 min, mixed with 900 µl SM buffer and centrifuged at 8500 × g for 1 min. The supernatant was filtered by a 0.22 μm filter, and the phage titer was measured by DLA method. The burst size was calculated as the ratio of the final number of phage particles to the initial number of infected host cells at the beginning of the test.

### Phage genome extraction and analysis

In this study, we used the randomly amplified polymorphic DNA (RAPD)-PCR technique described previously [36] for rapid screening and typing of *Escherichia* phage AG- MK-2022.Basu. Before the RAPD-PCR assay, genomic DNA was extracted from purified phage at the highest concentration using a phage DNA isolation kit (Norgen Biotek Corp., Thorold, ON, Canada) following the manufacturer’s instructions. The extracted DNA purity and concentration were assayed by gel electrophoresis and a NanoDrop spectrometer (Thermo Scientific™ NanoDrop 2000, Waltham, MA, USA). RAPD-PCR amplification was performed using P1 (5′-CCGCAGCCAA-3′) and P2 (5′-AACGGGCAGA-3′) primers [36]. Master mix preparation and thermal conditions were done according to the previously described protocol [25] in the SimpliAmp™ thermal cycler (Thermo Fisher Scientific, Waltham, MA, USA). In each run, *Escherichia* phage VaT-2019a isolate PE17 and distilled deionized water were used as positive and negative controls. In addition, extracted phage DNA was investigated for the presence of the *E. coli* virulence associated genes (VAGs) *stx1*, *stx2*, and *hylA*, using the multiplex PCR method, as described previously [24]. Oligonucleotides sequences for VAGs detection were from previously published study [37]. Primers sequences, expected amplicon sizes, and thermal cycling conditions are presented in supplementary Table S1. *E. coli* serotype O157: H7 ATCC 43,895 harboring *stx1*, *stx2* and *hylA* genes were used as positive controls for the PCR. PCR-amplified products were analyzed in 0.8% (w/v) agarose gel (SinaClon, Iran) and electrophoresed at 110 V for 55 min. The gels were visualized under UV light and photographed using a UV Imager (Transilluminator, Vilber Lourmat, France). Moreover, the presence of mobile genetics elements (MGEs) i.e. class 1 (*intI1*) and class 2 (*intI2*) integrons, and antibiotic resistance genes (ARGs) associated with resistance to β-lactams (*bla*_TEM_), tetracycline (*tetA*), plasmid-mediated quinolone (*qnrA*), sulfonamide (*sul1*), and trimethoprim(*dfrA1*) were assessed in *Escherichia* phage AG- MK-2022.Basu using the PCR method. The primers used, and thermal cycling conditions are given in supplementary Table S1. The previously described procedure was used for master mix preparation and gel electrophoresis [7].

### Phage bacteriolytic activity

In vitro bacteriolytic activity of *Escherichia* phage AG- MK-2022. Basu was determined using the bacterial growth reduction assay method as previously described [38–40]. In brief, an overnight culture of *E. coli* ATCC 25,922 was inoculated in BHI broth and incubated at 37 °C with shaking at 180 rpm until the optical density at 600 nm (OD600 nm) reached 1 (early exponential growth phase). Then, 200 µl of bacterial culture was mixed with 100 µl of phage stock solution (10^9^ PFU/mL), and this mixture was incubated at 37 °C for 24 h. The samples were collected after 0, 2, 4, 6, 8, 10, 12 and 24 h of incubation, and bacterial growth was monitored by measurement of OD 600 nm using a Shimadzu (Kyoto, Japan) mini UV 1240 spectrophotometer. All assays were performed in triplicate, and in each experiment, a bacterial control (BC) and a phage control (PC) were used. The bacterial control was inoculated only with bacteria, and the phage control was inoculated with phages only.

### Phage bactericidal activity against avian pathogenic *E. Coli*

In vitro bactericidal potential of both phages (*Escherichia* phage VaT-2019a isolate PE17 and *Escherichia* phage AG- MK-2022. Basu) against APEC strains (*n* = 100) were estimated by double agar overlay plaque assay [41], spot method, and bacterial growth reduction assay procedure as described above. During these experiments, the titer of phage was 10^9^ PFU/ml, and APEC strains at exponential growth phase (OD 600 nm = 1) were used instead of the host bacteria. The APEC strains were isolated from broiler chickens with clinical signs of colibacillosis on eight different broiler farms in Hamedan, western Iran. The sampling details, bacterial isolation and identification methods, antibiotic resistance profile, and MDR properties of these strains were described earlier [7, 24].

### Statistical analysis

Independent *t*-test was used to compare bacterial growth reduction rate (section Phage bactericidal activity against avian pathogenic *E. coli*) in the presence and absence of the phages. In addition, statistical analysis of the results of the bacteriophages infection test were performed by independent *t*-test and, the mean logarithm of the number of bacteria in the two groups. The IBM SPSS Statistics software version 26 programs for Windows (IBM Corp, Armonk, NY) was used for statistical analysis.

## Results

### Phage isolation and purification

**Fig. 1:**
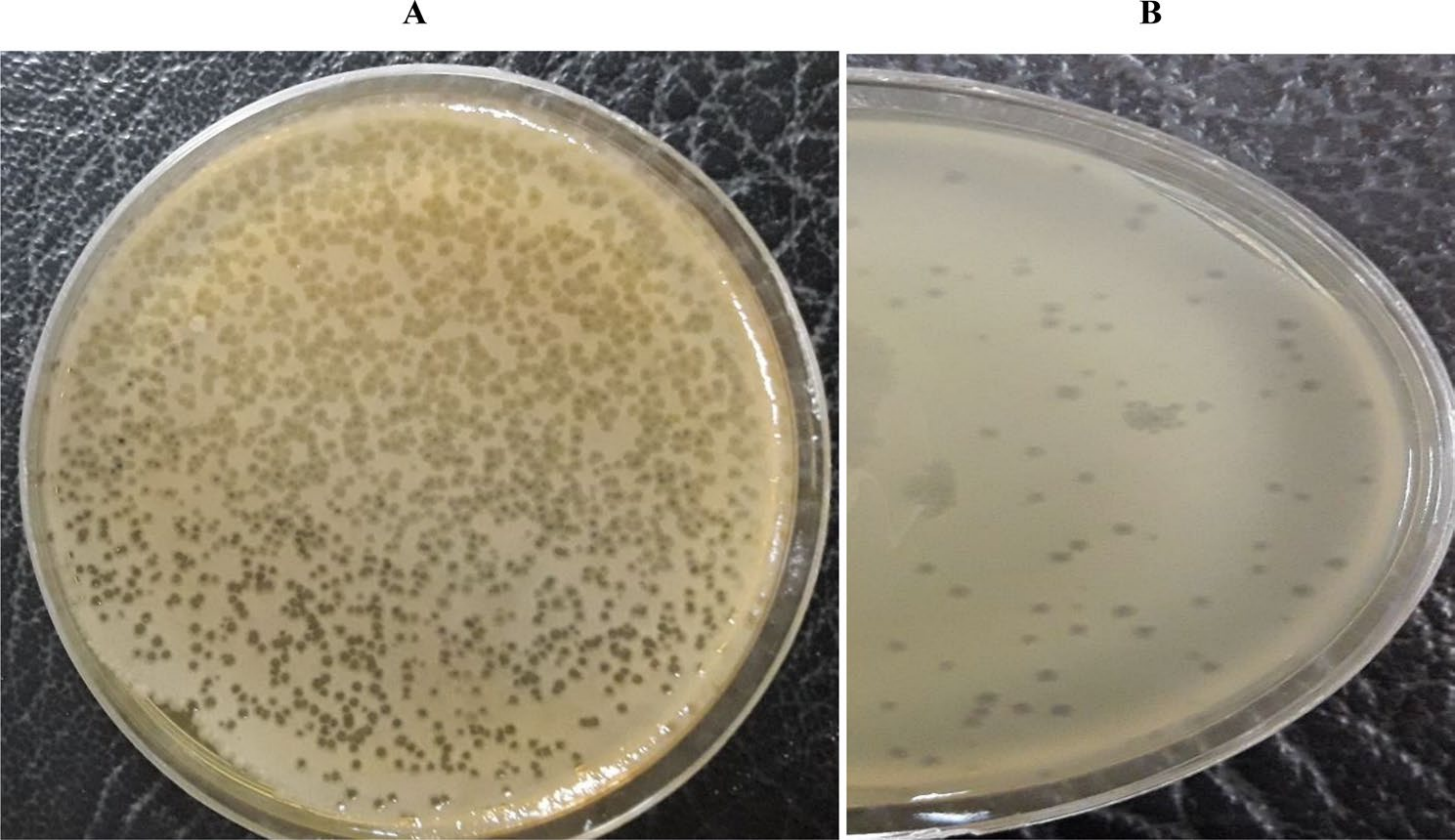
The plaques of *Escherichia* phage AG- MK-2022.Basu formed on a double-layered agar plate. **A**: 10^9^PFU/mL. **B**: 10^3^ PFU/mL

*Escherichia* phage AG- MK-2022. Basu was isolated from poultry slaughterhouse sewage water samples in Hamedan, Iran, using *E. coli* ATCC 25,922 as a host. This phage formed large, clear plaques specifically in the presence of a host strain with a diameter of 1–4 millimeters (Fig. 1). Here, we describe the characterization of this bacteriophage.

### Bacteriophage morphology

**Fig. 2:**
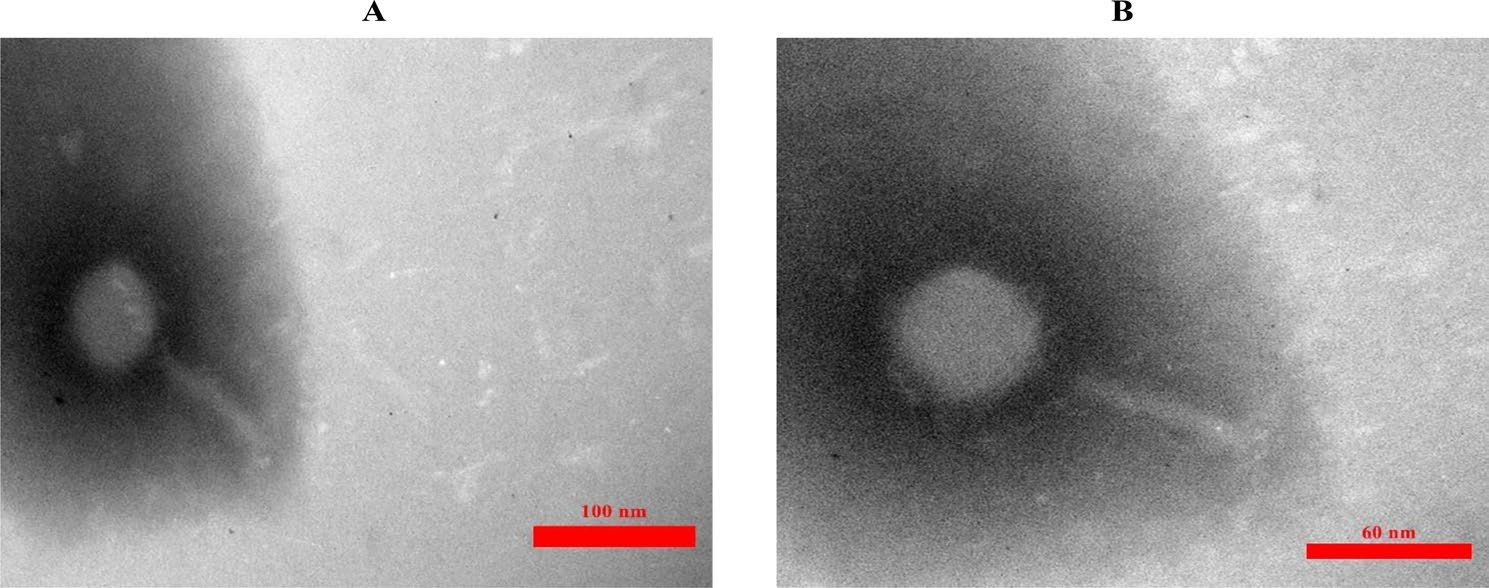
Transmission electron microscopy (TEM) of the *Escherichia* phage AG- MK-2022.Basu. **A**: Scale bar, 100 nm (Magnification = X50000) and **B**: Scale bar, 60 nm (Magnification = X85000)

Transmission electron microscopy (TEM) revealed that *Escherichia* phage AG- MK-2022. Basu had a tail (*caudovirales*) and structural characteristics similar to phages of the family *Myoviridae* (Fig. 2).

### Phage host range and EOP

The host range of *Escherichia* phage AG- MK-2022. Basu was estimated by spot test. Four out of seven tested strains (57.1%) showed a clear plaque in a spot assay test, including two *E. coli* strains, Salmonella and *Pseudomonas aeruginosa* (Table 1).

The EOP assay was performed for positive bacteria in the spot test, and the results of the EOP test indicate that the *Escherichia* phage AG- MK-2022. Basu showed high, moderate, and low efficiency in 1, 2 and 1 strains, respectively (Table 2).

**Table 2:** Host range and efficiency of plating (EOP) of the *Escherichia* phage AG- MK-2022. Basu

Bacteria	EOP^1^	Effect of phage aganists bacterial
*Escherichia coli* ATCC 25,922	1.2	High
*Escherichia coli* serotype O157: H7 ATCC 43,895	0.34	Moderate
*Salmonella enterica* subsp. *enterica serovar Typhimurium* ATCC 14,028	0.014	Low
*Pseudomonas aeruginosa* ATCC 27,253	0.27	Moderate

^1^Efficiency of plating

### Phage stability

**Fig. 3:**
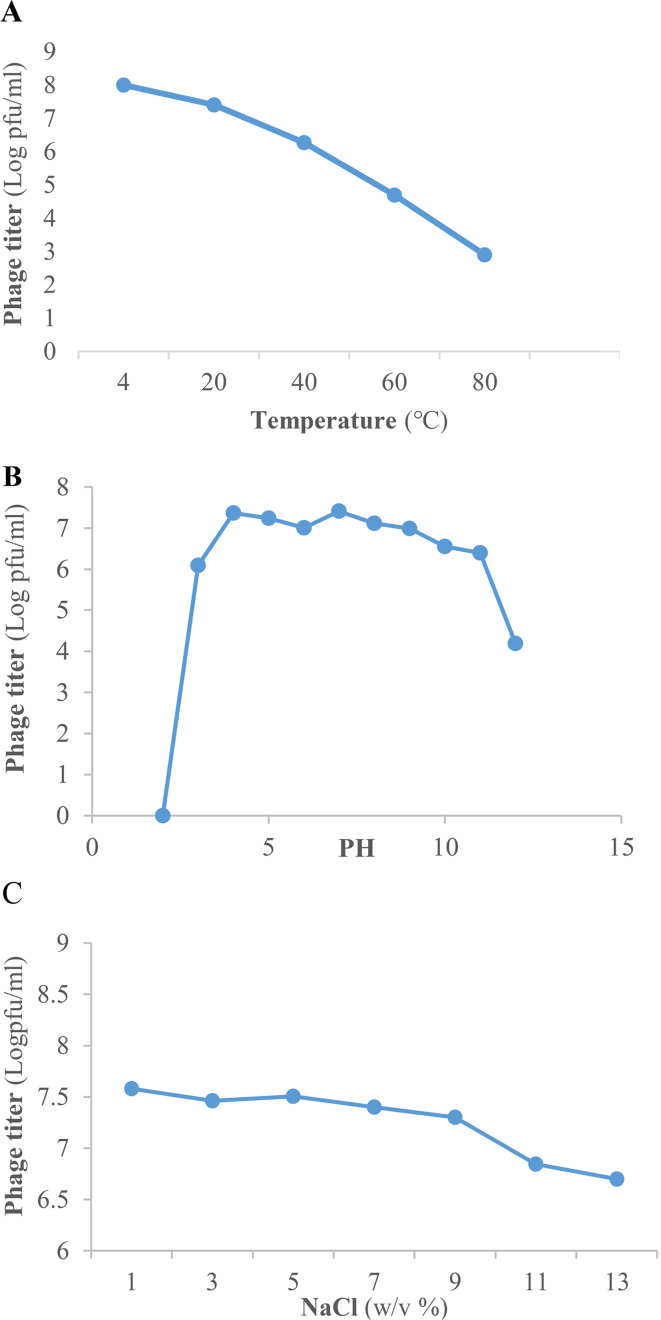
*Escherichia* phage AG- MK-2022. Basu stability in various conditions: **(A**) temperature; (**B**) pH; (**C**) NaCl

The results of *Escherichia* phage AG- MK-2022. Basu stability under various conditions, including temperature, pH, and NaCl concentration, is demonstrated in Fig. 3. The heat stability results revealed that the *Escherichia* phage AG- MK-2022. Basu survived at 4 °C to 80 °C, and there was no significant reduction in phage titer after incubation at temperatures between 4 °C and 40 °C, but the phage titer dropped significantly to 4.6 log PFU/ml at 60 °C and to 2.9 log PFU/ml at 80 °C compared to 4 °C. However, the phage could survive even incubation at 80 °C, but the lytic activity of phage was significantly reduced. Additionally, the phage remained stable when stored at 4 °C for four months (Fig. 3-A). As shown in Fig. 3-B, there was no significant difference in phage titer after 1 h incubation at pH 4–10. None of the phages survived at pH 2; however, at pH = 3 and 12, a decrease in phage titer was observed compared to standard conditions (pH = 7). There was no significant reduction in phage titer in the 1–9% NaCl range; however, increasing the NaCl concentration to 11% and 13% caused a significant drop in phage titer by 0.73 and 0.88 log PFU/ml, respectively, compared to the 1% concentration of NaCl (Fig. 3-C).

### Phage adsorption assay and one-step growth curve

**Fig. 4:**
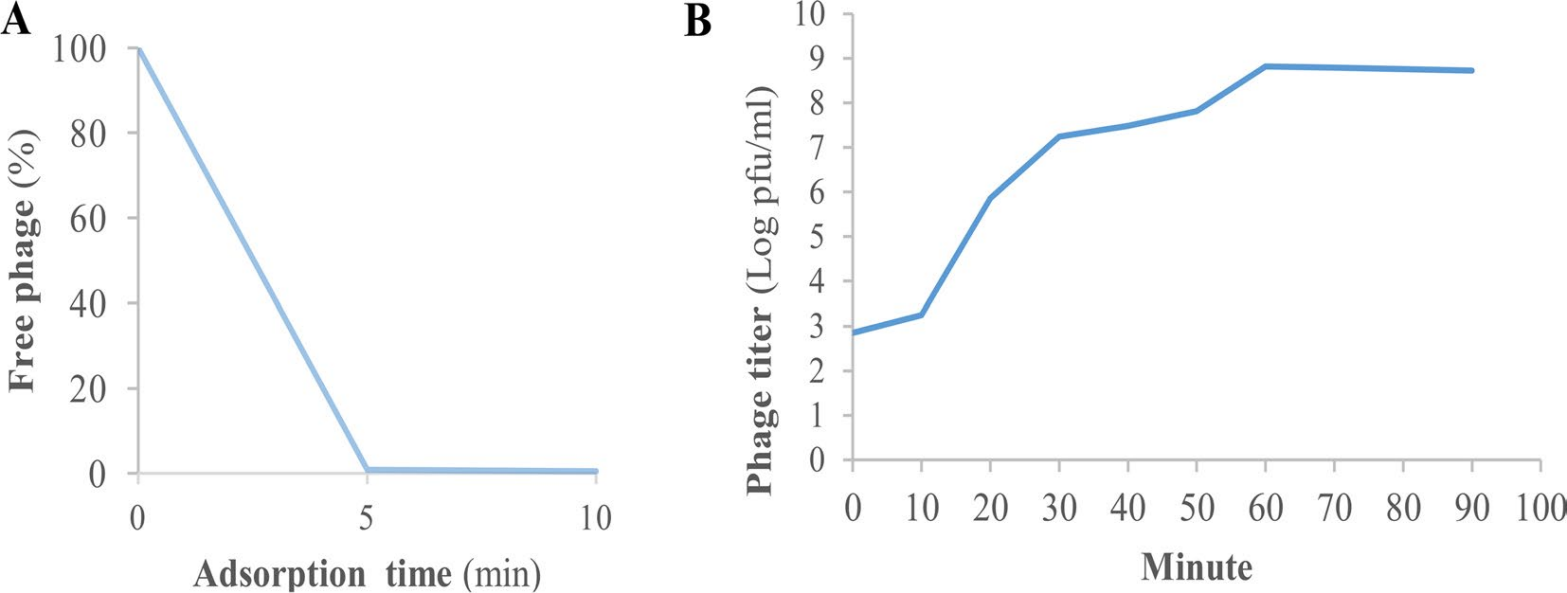
Adsorption of *Escherichia* phage AG- MK-2022. Basu to *E. coli* ATCC 25,922 **(A)**; One-step growth curves of *Escherichia* phage AG- MK-2022. Basu in the presence of *E. coli* ATCC 25,922 as host **(B)**

Phage adsorption assay showed that approximately 99.2% of the *Escherichia* phage AG- MK-2022. Basu particles adsorbed to the host bacterial cells after 5 min (Fig. 4-A). One-step growth curve revealed that *Escherichia* phage AG- MK-2022. Basu had a latent period of 10 min and a burst size of 152 PFU / host cell (Fig. 4-B).

### Genome analysis

**Fig. 5:**
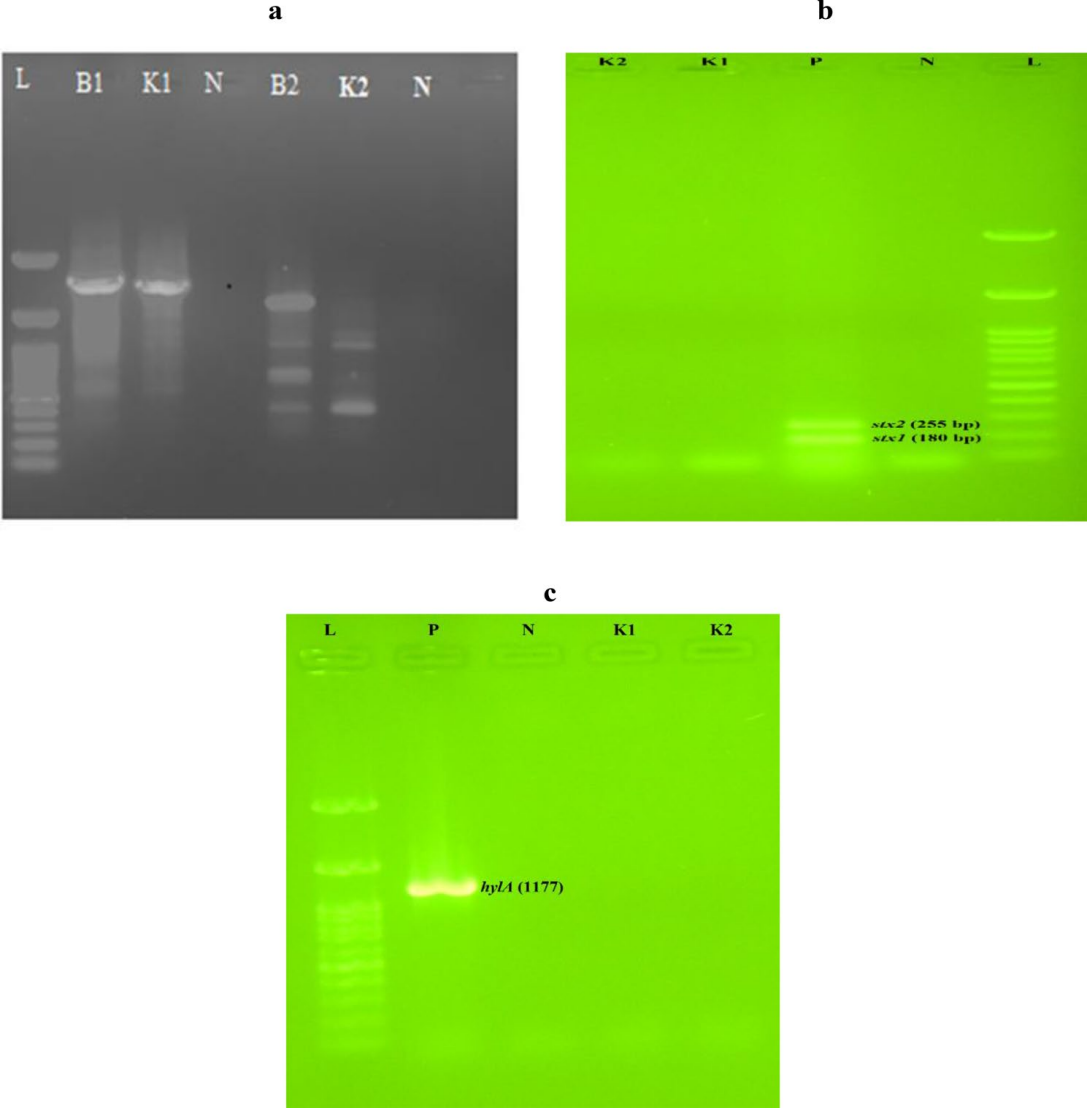
**(a)** RAPD band patterns obtained from *Escherichia* phage VaT-2019a isolate PE17(B1 and B2) and, *Escherichia* phage AG- MK-2022.Basu (K1 and K2) using P1 and P2 primers, respectively; (**b**) and (**c**) PCR for detection of *Escherichia coli* virulence associated genes *stx1*, *stx2*, and *hylA*. (L) marker 100 bp, (N) negative control, (P) positive control (*Escherichia coli* O157: H7 ATCC 43,895), (B1) *Escherichia* phage VaT-2019a isolate PE17 with primer P1, (K1) *Escherichia* phage AG- MK-2022.Basu with primer P1, (B2) *Escherichia* phage VaT-2019a isolate PE17 with primer P2, (K2) *Escherichia* phage AG- MK-2022.Basu with primer P2

As shown in Fig. 5-a, RAPD band patterns with amplicon sizes in the range of 1000–1500 bp with primer P1 and two distinct band patterns with amplicons ranging in size from 400 to 500 bp and 700–800 bp with primer P2 were observed after gel electrophoresis. Pure DNA of *Escherichia* phage VaT-2019a isolate PE17 was used as a control (Fig. 5-a). By applying RAPD PCR, it is also possible to discriminate between different phage lineages, with no need for whole genome sequencing [18, 36]. The PCR results for VAGs revealed that *E*. *coli* virulence associated genes (*stx1*, *stx2*, and *hylA*) were not detected in *Escherichia* phage AG- MK-2022. Basu (Fig. 5-b and 5-c). The PCR results for ARGs and MGEs showed that the *Escherichia* phage AG- MK-2022. Basu does not contain genes encoding antibiotic resistance (*bla*_TEM_, *tetA*, *qnrA*, *sul1*, and *dfrA1*), and integrons (*intI1* and *intI2*).

### Phage bacteriolytic activity

**Fig. 6:**
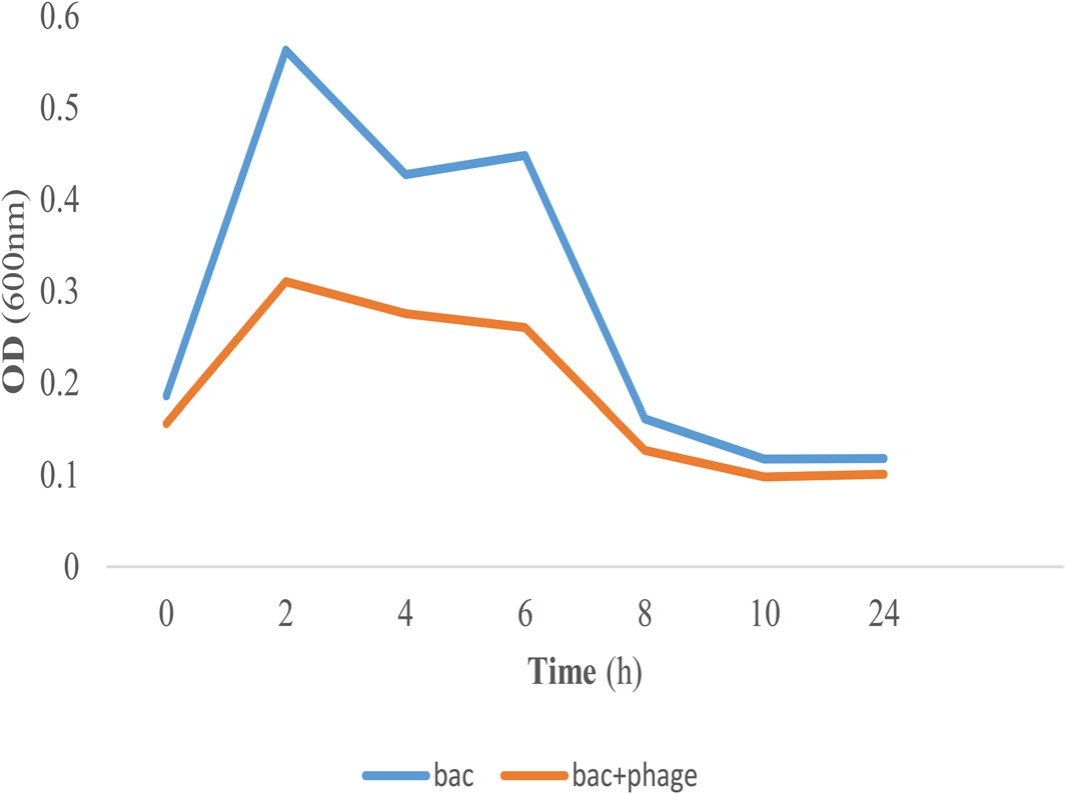
Bacterial challenge test of phage *Escherichia* phage AG- MK-2022. with *Escherichia*. coli ATCC 25,922. Bac + phage: bacteria + phage, bac: bacteria

A bacterial growth reduction assay was used to evaluate the lytic activity of *Escherichia* phage AG- MK-2022. Basu against the host strain (*E. coli* ATCC25922) in vitro. The results revealed that *Escherichia* phage AG- MK-2022. Basu exhibited strong lytic activity against their original host (*E. coli* ATCC25922) in vitro, and significant decreases in the viability of bacterial strains were observed after phage administration (MOI = 1) compared to the bacterial control (Fig. 6).

### Phage bactericidal activity against avian pathogenic *E. Coli*

**Fig. 7:**
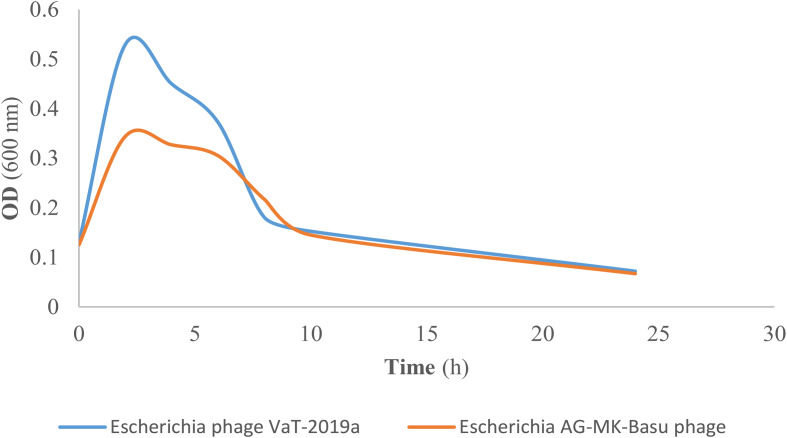
Bacteriolytic activity of *Escherichia* phage AG- MK-2022. Basu and, *Escherichia* phage VaT-2019a isolate PE17 against Avian Pathogenic *Escherichia coli* (APEC) strains in vitro

Tables S1 and S2 describes the in vitro bacteriolytic activity of both phages (*Escherichia* phage VaT-2019a isolate PE17 and *Escherichia* phage AG- MK-2022. Basu) against APEC strains (*n* = 100) by double agar overlay plaque assay, spot test, and bacterial growth reduction assay. As demonstrated in Table S2, *Escherichia* phage AG- MK-2022 and *Escherichia* phage VaT-2019a isolate PE17 lysed 95 (95%) of the 100 APEC strains in the spot assay test (Table S2). In addition, the in vitro bacterial growth reduction assay results revealed that both phages cause significant decreases in the viability of 95% of the examined APEC strains (OD 600 nm) six hours after phage addition compared with the control group (Fig. 7 and Tables S1 and S2). The double agar overlay plaque assay results indicate that most APEC strains (45%) were susceptible to phage dilution at 10^− 1^ (Table 3).

**Table 3:** Phages bactericidal activity against avian pathogenic *Escherichia coli* (APEC) in different dilutions of phages

Dilution of phages	Number of susceptible APEC strains (*n* = 100)	
	*Escherichia* phage VaT-2019a isolate PE17	*Escherichia* phage AG-MK-Basu.2022
10^− 1^	45	44
10^− 2^	28	26
10^− 3^	16	13
10^− 4^	5	12
10^− 5^	6	5

### Statistical analysis results

A significant decrease was observed for the OD values at 600 nm in 95 / 100 of tested APEC strains in the presence of phages compared to the mean values in the absence of the phages (Table S2).

## Discussion

Recently, the emergence of multidrug-resistant bacterial pathogens highlights the need for new strategies to combat them and has demonstrated the value of phages as antibacterial agents for medical and veterinary applications [17, 32]. In addition, MDR-APEC strains pose a significant threat to poultry health and lead to economic losses in the poultry industry [7, 22, 42–44], emphasizing the need for alternative approaches for decreasing the incidence of MDR-APEC strains in poultry [45].

In this study, the new phage (*Escherichia* phage AG- MK-2022. Basu) was isolated from poultry slaughterhouse sewage and selected because it has high host specificity to *E. coli* strains. Several studies have reported the isolation of lytic specific phages for *E. coli* from poultry sewage samples, and these findings confirm that sewage samples are rich sources for isolating lytic bacteriophages [35, 46, 47].

The morphological characteristics of the isolated phage observed by transmission electronic microscopy (TEM) indicate that *Escherichia* phage AG- MK-2022. Basu belong to the order *Caudovirales* and family *Myoviridae.* The same phage types have been isolated from sewage and morphologically characterized previously [25, 47, 48].

Host range determination findings revealed that the *Escherichia* phage AG- MK-2022. Basu not only lysed the host bacterium but also showed lytic activity against four out of the seven tested bacteria, which were all Gram-negative bacteria, so this phage is a broad-host-range phage with the ability to infect three different genera (*Escherichia*, *Salmonella* and *Pseudomonas*) and may have a polyvalent nature. Previous studies have also reported the isolation and identification of polyvalent *Escherichia* phages that are able to lyse wide ranges of Gram-negative bacteria simultaneously [25, 28, 49–52]. However, the results of the EOP test indicated that *Escherichia* phage AG- MK-2022. Basu was highly specific and effective against *E*. *coli* strains. Similar findings were reported in earlier studies that introduced broad host range *Escherichia* phages that were extremely effective against *E. coli* strains [48, 53, 54].

Bacteriophage survival under different conditions is one of the most important criteria in the selection of suitable phages to be used in phage therapy [28]. *Escherichia* phage AG- MK-2022. Basu exhibited stability at a wide range of temperatures (4–80 °C), pH values (4–10), and NaCl concentrations (1–13%). These findings are in accordance with prior investigations [33, 55], which have isolated and identified highly stable *Escherichia* phages. The stability of phage in these conditions indicates that this phage could be used in a variety of environmental conditions.

In the current study, genome analysis of the *Escherichia* phage AG- MK-2022. Basu revealed that the isolated phage genome possesses no antibiotic resistance genes (ARGs), mobile genetic elements (MGEs), and *E*. *coli* virulence associated genes (VAGs) assessed. These findings was in accordance with previous studies [25, 30, 54, 56] which reported that *Escherichia* phage genome did not harbor any genes associated with pathogenicity and distribution of antibiotic resistance. These findings indicate that the phage may be safely used as a biocontrol agent, however more work, such as whole genome sequencing of the isolated phage, is necessary to achieved more information regarding its safe use.

In recent years, there has been growing interest in phage therapy as an effective therapeutic tool against colibacillosis caused by APEC due to the increasing number of multidrug-resistant APEC strains [54, 57, 58]. The in vitro bacterial challenge tests demonstrated that two phages, *Escherichia* phage VaT-2019a isolate PE17 and *Escherichia* phage AG- MK-2022. Basu was highly effective and exhibited high bactericidal activity against the APEC strains we tested. Furthermore, these phages could be used as an alternative to antibiotics for controlling MDR-APEC strains isolated from broilers. Previous studies reported the characterization and application of bacteriophages against APEC strains of poultry origin. A very recent study from Pakistan, Sattar et al. [40]. reported the isolation, characterization, and genome analysis of two lytic phages (*Escherichia* phage SKA49 and *Escherichia* phage SKA64) against the MDR strain of APEC and stated that these phages can be good candidates for the control of APEC strains. Zhang et al. [59] introduced phage Bp7 as a wide host range phage and suggested its polyvalent nature and as an alternative to antimicrobials for controlling drug-resistant *E. coli* in chickens [59]. Tang et al. [54] isolated a newly lytic phage, CE1, from broiler feces. Evaluation of the bactericidal activity of this phage against APEC strains in vitro and in vivo confirmed that the phage has a broad host range and lysed 56.9% (33/58) of highly pathogenic strains of APEC. Nicolas et al. [60] isolated and characterized 19 novel phage collections against APEC strains and reported a cocktail of eight phages to be a promising candidate for the biocontrol of avian colibacillosis. In another study, Kazibwe et al. [45] described seven lytic bacteriophages against APEC and reported that two phages, UPEC04 and UPEC10, had combined lytic activity against APEC strains isolated from broilers. A study performed by Zhou et al. [48] isolated and described JS09 phage from sewage, which was able to infect some clinically isolated antibiotic-resistant APEC and ETEC strains and suggested the phage as a candidate for phage therapy to control colibacillosis in animals.

There were some limitations in the present study such as limitation in whole genome sequencing of newly isolated phage (*Escherichia* phage AG- MK-2022. Basu) because of resources. Moreover, whole genome sequencing could provide insightful information about the newly isolated phage regarding its safe use.

## Conclusion

In conclusion, this study confirmed two novel phages, the *Escherichia* phage VaT-2019a isolate PE17 and the *Escherichia* phage AG- MK-2022. Basu exhibited strong antimicrobial effects against APEC strains in vitro and could be suggested as novel biocontrol agents to combat the occurrence of MDR-APEC strains. In vivo evaluations will be suggested to achieve a better picture of probable therapeutic/biocontrol applications of these phages in poultry farms.

### Electronic supplementary material

Below is the link to the electronic supplementary material.

Supplementary Material 1

## Data Availability

The datasets generated and analysed during the current study are available in the NCBI—GenBank—Nucleotide platform (https://www.ncbi.nlm.nih.gov/genbank/) and can be accessed through accession number: GenBank: MK353636.1.
