# Supplementary material for: In vitro evaluation of two novel Escherichia bacteriophages against multiple drug resistant avian pathogenic Escherichia coli
Source: BMC Infect Dis. 2024 May 16;24:497. doi: 10.1186/s12879-024-09402-0 (PMC11100137; doi:10.1186/s12879-024-09402-0)
Supplement: Supplementary file 1 — Supplementary Material 1 [file 12879_2024_9402_MOESM1_ESM.docx]

Table **S1** The oligonucleotide primers and PCR conditions used for molecular detection of the *Escherichia* *coli* virulence associated genes, antibiotic resistance genes and mobile genetic elements in this study.

| **Primer** | **oligonucleotide sequence**  **(5'→3')** | **Target gene** | **Amplicon size**  **(bp)** | **Cycling conditions** |
| --- | --- | --- | --- | --- |
| **Virulence associated genes primers** | | | | |
| Stx1-F  Stx1-R | ATAAATCGCCATTCGTTGACTAC  AGAACGCCCACTGAGATCATC | *stx1* | 180 | 95 °C–1min; (×35) 95°C-1min, 65°C (for the first 10 cycles) and, 60°C (by cycle 15)- 2 min; 72°C- 1.5 min and, incrementing to 2.5 min from cycles 25 to 35. |
| stx2-F  stx2-R | GGCACTGTCTGAAACTGCTCC  TCGCCAGTTATCTGACATTCTG | s*tx2* | 255 |  |
| hlyA- F  hlyA- R | GCATCATCAAGCGTACGTTCC  AATGAGCCAAGCTGGTTAAGCT | *hlyA* | 1177 | 94 °C–3 min; (× 30) 94 °C–30 s, 58 °C–30 s, 68 °C–3 min; 72 °C–10 min |
| **Antibiotic resistance genes /integron primers** | | | | |
| Bla_TEM_ -F  Bla_TEM_ -R | GAGTATTCAACATTTTCGT  ACCAATGCTTAATCAGTGA | *bla_TEM_* | 857 | 94 °C– 4 min; (× 30) 94 °C–30 s, 50 °C–40 s, 72 °C– 1 min; 72 °C–10 min |
| tetA-F  tetA-R | GTGAAACCCAACATACCCC  GAAGGCAAGCAGGATGTAG | *tetA* | 888 | 94 °C– 4 min; (× 30) 94 °C–30 s, 50 °C–40 s, 72 °C–1 min; 72 °C–10 min |
| qnrA -F  qnrA -R | ATTTCTCACGCCAGGATTTG  GATCGGCAAAGGTTAGGTCA | *qnrA* | 516 | 94 °C– 4 min; (× 30) 94 °C–30 s, 53 °C–40 s, 72 °C– 1 min; 72 °C–10 min |
| dfrA1-F  dfrA1-F | CCCAACCGAAAGTATGCGGTCG  GTATCTACTTGATCGATCAGG | *dfrA1*-*like* | 171 | 94 °C– 4 min; (× 30) 94 °C–30 s, 50 °C–40 s, 72 °C– 1 min; 72 °C–10 min |
| Sul1-F  Sul1-R | CGGCGTGGGCTACCTGAACG  GCCGATCGCGTGAAGTTCCG | *sul1* | 433 | 94 °C– 4 min; (× 30) 94 °C–30 s, 50 °C–40 s, 72 °C– 1 min; 72 °C–10 min |
| Int1-F  Int1-R | GGGGTCAAGGATCTGGATTTCG  ACATGCGTGTAAATCATCGTCG | *intI1* | 485 | 94 °C– 4 min; (× 30) 94 °C–30 s, 62.5 °C–40 s, 72 °C– 1 min; 72 °C–8 min |
| Int2-F  Int2-R | CACGGATATGCGACAAAAAGGT  GTAGCAAACGAGTGACGAAATG | *intI2* | 790 |  |

**Table S2** *In vitro* bacteriolytic activity of *Escherichia* phage VaT-2019a isolate PE17 and, *Escherichia* phage AG- MK-2022.Basu against Avian Pathogenic *Escherichia coli* (APEC) strains (*n*= 100)

| **APEC strain Id** | ***Escherichia*** **phage VaT-2019a** | | | ***Escherichia* phage AG-MK-Basu.2022** | | |
| --- | --- | --- | --- | --- | --- | --- |
|  | **Spot test** | **Bacterial growth reduction assay (mean decrease of OD 600 nm)** | **Double agar overlay plaque assay (PFU/ml)** | **Spot test** | **Bacterial growth reduction assay (mean decrease of OD 600 nm)** | **Double agar overlay plaque assay (PFU/ml)** |
| L11 | + | 0.1765 | 2*10^4^ | + | 0.124 | 10^3^ |
| L26 | + | 0.1325 | 10^3^ | + | 0.085 | 2*10^4^ |
| A11 | + | 0.127 | 2*10^4^ | + | 0.106 | 3*10^5^ |
| A33 | + | 0.1665 | 103 | + | 0.123 | 10^3^ |
| L14 | + | 0.1075 | 2*10^4^ | + | 0.125 | 10^3^ |
| A89 | + | 0.176 | 3*10^5^ | + | 0.163 | 5*10^7^ |
| L99 | + | 0.191 | 2*10^4^ | + | 0.157 | 2*10^4^ |
| C37 | + | 0.223 | 2*10^4^ | + | 0.145 | 10^3^ |
| L3 | + | 0.273 | 10^3^ | + | 0.242 | 10^3^ |
| C43 | + | 0.138 | 10^3^ | + | 0.159 | 10^3^ |
| L13 | + | 0.1805 | 0 | + | 0.145 | 10^3^ |
| T74 | + | 0.192 | 3*10^5^ | + | 0.145 | 10^3^ |
| T9 | + | 0.162 | 0 | + | 0.139 | 2*10^4^ |
| A25 | + | 0.1135 | 2*10^4^ | + | 0.1155 | 10^3^ |
| C40 | + | 0.1365 | 2*10^4^ | + | 0.197 | 10^3^ |
| C33 | + | 0.302 | 2*10^4^ | + | 0.138 | 2*10^4^ |
| A7 | - |  | 0 | - |  | 0 |
| L94 | - |  | 0 | - |  | 0 |
| T28 | + | 0.343 | 3*10^5^ | + | 0.1165 | 3*10^5^ |
| T22 | + | 0.154 | 2*10^4^ | + | 0.152 | 10^3^ |
| L118 | + | 0.1945 | 2*10^4^ | + | 0.095 | 3*10^5^ |
| A45 | + | 0.1115 | 10^3^ | + | 0.107 | 2*10^4^ |
| T105 | + | 0.257 | 10^3^ | + | 0.124 | 10^3^ |
| T49 | + | 0.212 | 2*10^4^ | + | 0.155 | 2*10^4^ |
| T16 | + | 0.145 | 5*10^7^ | + | 0.145 | 2*10^4^ |
| C34 | + | 0.167 | 3*10^5^ | + | 0.1705 | 4*10^6^ |
| C108 | + | 0.1525 | 3*10^5^ | + | 0.186 | 2*10^4^ |
| L5 | + | 0.3505 | 10^3^ | + | 0.089 | 2*10^4^ |
| C11 | + | 0.1315 | 3*10^5^ | + | 0.168 | 2*10^4^ |
| L34 | + | 0.2205 | 2*10^4^ | + | 0.195 | 4*10^6^ |
| T33 | + | 0.147 | 10^3^ | + | 0.194 | 10^3^ |
| L76 | + | 0.1585 | 3*10^5^ | - |  | 0 |
| L52 | + | 0.163 | 10^3^ | + | 0.127 | 2*10^4^ |
| L29 | + | 0.138 | 10^3^ | + | 0.152 | 0 |
| T34 | + | 0.1 | 2*10^4^ | + | 0.117 | 3*10^5^ |
| **APEC strain Id** | ***Escherichia*** **phage VaT-2019a** | | | ***Escherichia* phage AG-MK-Basu.2022** | | |
|  | **Spot test** | **Bacterial growth reduction assay (mean decrease of OD 600 nm)** | **Double agar overlay plaque assay (PFU/ml)** | **Spot test** | **Bacterial growth reduction assay (mean decrease of OD 600 nm)** | **Double agar overlay plaque assay (PFU/ml)** |
| C4 | + | 0.124 | 3*10^5^ | + | 0.125 | 2*10^4^ |
| L100 | + | 0.128 | 2*10^4^ | + | 0.125 | 4*10^6^ |
| C2 | + | 0.143 | 2*10^4^ | + | 0.186 | 4*10^6^ |
| C7 | + | 0.119 | 3*10^5^ | + | 0.198 | 3*10^5^ |
| L36 | + | 0.196 | 4*10^6^ | + | 0.1625 | 2*10^4^ |
| L46 | + | 0.115 | 10^3^ | + | 0.154 | 4*10^6^ |
| T11 | + | 0.125 | 2*10^4^ | + | 0.1175 | 2*10^4^ |
| L23 | + | 0.1795 | 10^3^ | + | 0.127 | 10^3^ |
| A36 | + | 0.177 | 10^3^ | + | 0.267 | 10^3^ |
| L69 | + | 0.1555 | 3*10^5^ | + | 0.158 | 4*10^6^ |
| A12 | + | 0.2095 | 2*10^4^ | + | 0.1726 | 10^3^ |
| T47 | + | 0.171 | 10^3^ | + | 0.264 | 2*10^4^ |
| L10 | + | 0.152 | 10^3^ | + | 0.169 | 10^3^ |
| L120 | + | 0.153 | 5*10^7^ | + | 0.1135 | 5*10^7^ |
| L104 | + | 0.136 | 2*10^4^ | + | 0.104 | 2*10^4^ |
| T17 | + | 0.138 | 2*10^4^ | + | 0.245 | 3*10^5^ |
| T12 | + | 0.049 | 10^3^ | + | 0.124 | 2*10^4^ |
| L2 | + | 0.126 | 3*10^5^ | + | 0.243 | 10^3^ |
| A10 | + | 0.1335 | 10^3^ | + | 0.247 | 10^3^ |
| A77 | + | 0.0855 | 2*10^4^ | + | 0.145 | 3*10^5^ |
| C44 | + | 0.147 | 10^3^ | + | 0.196 | 10^3^ |
| T90 | + | 0.0835 | 10^3^ | + | 0.1075 | 4*10^6^ |
| C98 | - |  | 0 | - |  | 0 |
| C18 | + | 0.08 | 10^3^ | + | 0.256 | 3*10^5^ |
| T10 | + | 0.0715 | 2*10^4^ | + | 0.171 | 2*10^4^ |
| T23 | + | 0.116 | 2*10^4^ | + | 0.153 | 2*10^4^ |
| A28 | + | 0.085 | 2*10^4^ | + | 0.2265 | 10^3^ |
| A2 | + | 0.171 | 10^3^ | + | 0.199 | 3*10^5^ |
| T40 | + | 0.1405 | 10^3^ | + | 0.175 | 5*10^7^ |
| T119 | + | 0.124 | 2*10^4^ | + | 0.12 | 10^3^ |
| T69 | + | 0.156 | 4*10^6^ | + | 0.1985 | 10^3^ |
| C14 | + | 0.169 | 10^3^ | + | 0.233 | 3*10^5^ |
| T89 | - |  | 0 | - |  | 0 |
| T6 | + | 0.152 | 10^3^ | + | 0.246 | 10^3^ |
| C13 | + | 0.1505 | 3*10^5^ | + | 0.184 | 10^3^ |
| L39 | + | 0.092 | 10^3^ | + | 0.132 | 3*10^5^ |
| T114 | + | 0.11 | 10^3^ | + | 0.145 | 10^3^ |
| C10 | + | 0.126 | 3*10^5^ | + | 0.145 | 2*10^4^ |
| A31 | + | 0.145 | 10^3^ | + | 0.178 | 10^3^ |
| **APEC strain Id** | ***Escherichia*** **phage VaT-2019a** | | | ***Escherichia* phage AG-MK-Basu.2022** | | |
|  | **Spot test** | **Bacterial growth reduction assay (mean decrease of OD 600 nm)** | **Double agar overlay plaque assay (PFU/ml)** | **Spot test** | **Bacterial growth reduction assay (mean decrease of OD 600 nm)** | **Double agar overlay plaque assay (PFU/ml)** |
| C42 | + | 0.133 | 10^3^ | + | 0.129 | 2*10^4^ |
| L20 | + | 0.15 | 2*10^4^ | + | 0.1185 | 2*10^4^ |
| T5 | + | 0.127 | 4*10^6^ | + | 0.243 | 10^3^ |
| L8 | + | 0.0915 | 5*10^7^ | + | 0.136 | 2*10^4^ |
| T25 | + | 0.119 | 10^3^ | + | 0.195 | 10^3^ |
| A34 | + | 0.191 | 10^3^ | + | 0.214 | 10^3^ |
| A100 | + | 0.0885 | 10^3^ | + | 0.247 | 4*10^6^ |
| A105 | + | 0.1095 | 2*10^4^ | + | 0.13 | 10^3^ |
| C35 | + | 0.054 | 10^3^ | + | 0.215 | 4*10^6^ |
| C26 | + | 0.196 | 10^3^ | + | 0.1 | 10^3^ |
| A3 | + | 0.146 | 3*10^5^ | + | 0.169 | 10^3^ |
| C119 | + | 0.3445 | 10^3^ | + | 0.555 | 10^3^ |
| T2 | + | 0.085 | 3*10^5^ | + | 0.135 | 2*10^4^ |
| C12 | + | 0.062 | 10^3^ | + | 0.247 | 10^3^ |
| A26 | + | 0.0665 | 10^3^ | + | 0.2395 | 10^3^ |
| C90 | + | 0.1954 | 10^3^ | + | 0.164 | 10^3^ |
| C45 | + | 0.062 | 10^3^ | + | 0.125 | 2*10^4^ |
| C53 | + | 0.063 | 3*10^5^ | + | 0.145 | 3*10^5^ |
| A14 | + | 0.085 | 5*10^7^ | + | 0.178 | 10^3^ |
| A13 | - |  | 0 | + | 0.123 | 10^3^ |
| T110 | + | 0.1375 | 10^3^ | + | 0.263 | 10^3^ |
| A16 | + | 0.065 | 10^3^ | + | 0.264 | 4*10^6^ |
| A107 | + | 0.0747 | 4*10^6^ | + | 0.185 | 4*10^6^ |
| A38 | + | 0.057 | 4*10^6^ | + | 0.1125 | 5*10^7^ |
| L83 | + | 0.071 | 5*10^7^ | + | 0.1795 | 10^3^ |
| C1 | + | 0.0715 | 2*10^4^ | + | 0.1545 | 2*10^4^ |
| A6 | + | 0.1135 | 10^3^ | + | 0.1085 | 10^3^ |
| T38 | + | 0.0755 | 2*10^4^ | + | 0.21 | 5*10^7^ |
| A42 | + | 0.0755 | 10^3^ | + | 0.195 | 4*10^6^ |
| A27 | + | 0.065 | 10^3^ | + | 0.12 | 10^3^ |
| L26 | + | 0.1325 | 10^3^ | + | 0.085 | 2*10^4^ |
| A11 | + | 0.127 | 2*10^4^ | + | 0.106 | 3*10^5^ |
| A33 | + | 0.1665 | 10^3^ | + | 0.123 | 10^3^ |
| L14 | + | 0.1075 | 2*10^4^ | + | 0.125 | 10^3^ |
| A89 | + | 0.176 | 3*10^5^ | + | 0.163 | 5*10^7^ |
| L99 | + | 0.191 | 2*10^4^ | + | 0.157 | 2*10^4^ |
| C37 | + | 0.223 | 2*10^4^ | + | 0.145 | 10^3^ |
| L3 | + | 0.273 | 10^3^ | + | 0.242 | 10^3^ |
| **APEC strain Id** | ***Escherichia*** **phage VaT-2019a** | | | ***Escherichia* phage AG-MK-Basu.2022** | | |
|  | **Spot test** | **Bacterial growth reduction assay (mean decrease of OD 600 nm)** | **Double agar overlay plaque assay (PFU/ml)** | **Spot test** | **Bacterial growth reduction assay (mean decrease of OD 600 nm)** | **Double agar overlay plaque assay (PFU/ml)** |
| C43 | + | 0.138 | 10^3^ | + | 0.159 | 10^3^ |
| L13 | + | 0.1805 | 0 | + | 0.145 | 10^3^ |
| T74 | + | 0.192 | 3*10^5^ | + | 0.145 | 10^3^ |
| T9 | + | 0.162 | 0 | + | 0.139 | 2*10^4^ |
| A25 | + | 0.1135 | 2*10^4^ | + | 0.1155 | 10^3^ |
| C40 | + | 0.1365 | 2*10^4^ | + | 0.197 | 10^3^ |
| C33 | + | 0.302 | 2*10^4^ | + | 0.138 | 2*10^4^ |
| A7 | - |  | 0 | - |  | 0 |
| L94 | - |  | 0 | - |  | 0 |
| T28 | + | 0.343 | 3*10^5^ | + | 0.1165 | 3*10^5^ |
| T22 | + | 0.154 | 2*10^4^ | + | 0.152 | 10^3^ |
| L118 | + | 0.1945 | 2*10^4^ | + | 0.095 | 3*10^5^ |
| A45 | + | 0.1115 | 10^3^ | + | 0.107 | 2*10^4^ |
| T105 | + | 0.257 | 10^3^ | + | 0.124 | 10^3^ |
| T49 | + | 0.212 | 2*10^4^ | + | 0.155 | 2*10^4^ |
| T16 | + | 0.145 | 5*10^7^ | + | 0.145 | 2*10^4^ |
| C34 | + | 0.167 | 3*10^5^ | + | 0.1705 | 4*10^6^ |
| C108 | + | 0.1525 | 3*10^5^ | + | 0.186 | 2*10^4^ |
| L5 | + | 0.3505 | 10^3^ | + | 0.089 | 2*10^4^ |
| C11 | + | 0.1315 | 3*10^5^ | + | 0.168 | 2*10^4^ |
| L34 | + | 0.2205 | 2*10^4^ | + | 0.195 | 4*10^6^ |
| T33 | + | 0.147 | 10^3^ | + | 0.194 | 10^3^ |
| L76 | + | 0.1585 | 3*10^5^ | - |  | 0 |
| L52 | + | 0.163 | 10^3^ | + | 0.127 | 2*10^4^ |
| L29 | + | 0.138 | 10^3^ | + | 0.152 | 0 |
| T34 | + | 0.1 | 2*10^4^ | + | 0.117 | 3*105 |
| C4 | + | 0.124 | 3*10^5^ | + | 0.125 | 2*10^4^ |
| L100 | + | 0.128 | 2*10^4^ | + | 0.125 | 4*10^6^ |
| C2 | + | 0.143 | 2*10^4^ | + | 0.186 | 4*10^6^ |
| C7 | + | 0.119 | 3*10^5^ | + | 0.198 | 3*10^5^ |
| L36 | + | 0.196 | 4*10^6^ | + | 0.1625 | 2*10^4^ |
| L46 | + | 0.115 | 10^3^ | + | 0.154 | 4*10^6^ |
| T11 | + | 0.125 | 2*10^4^ | + | 0.1175 | 2*10^4^ |
| L23 | + | 0.1795 | 10^3^ | + | 0.127 | 10^3^ |
| A36 | + | 0.177 | 10^3^ | + | 0.267 | 10^3^ |
| L69 | + | 0.1555 | 3*10^5^ | + | 0.158 | 4*10^6^ |
| A12 | + | 0.2095 | 2*10^4^ | + | 0.1726 | 10^3^ |
| T47 | + | 0.171 | 10^3^ | + | 0.264 | 2*10^4^ |
| L10 | + | 0.152 | 10^3^ | + | 0.169 | 10^3^ |
| **APEC strain Id** | ***Escherichia*** **phage VaT-2019a** | | | ***Escherichia* phage AG-MK-Basu.2022** | | |
|  | **Spot test** | **Bacterial growth reduction assay (mean decrease of OD 600 nm)** | **Double agar overlay plaque assay (PFU/ml)** | **Spot test** | **Bacterial growth reduction assay (mean decrease of OD 600 nm)** | **Double agar overlay plaque assay (PFU/ml)** |
| L120 | + | 0.153 | 5*10^7^ | + | 0.1135 | 5*10^7^ |
| L104 | + | 0.136 | 2*10^4^ | + | 0.104 | 2*10^4^ |
| T17 | + | 0.138 | 2*10^4^ | + | 0.245 | 3*10^5^ |
| T12 | + | 0.049 | 10^3^ | + | 0.124 | 2*10^4^ |
| L2 | + | 0.126 | 3*10^5^ | + | 0.243 | 10^3^ |
| A10 | + | 0.1335 | 10^3^ | + | 0.247 | 10^3^ |
| A77 | + | 0.0855 | 2*10^4^ | + | 0.145 | 3*10^5^ |
| C44 | + | 0.147 | 10^3^ | + | 0.196 | 10^3^ |
| T90 | + | 0.0835 | 10^3^ | + | 0.1075 | 4*10^6^ |
| C98 | - |  | 0 | - |  | 0 |
| C18 | + | 0.08 | 10^3^ | + | 0.256 | 3*10^5^ |
| T10 | + | 0.0715 | 2*10^4^ | + | 0.171 | 2*10^4^ |
| T23 | + | 0.116 | 2*10^4^ | + | 0.153 | 2*10^4^ |
| A28 | + | 0.085 | 2*10^4^ | + | 0.2265 | 10^3^ |
| A2 | + | 0.171 | 10^3^ | + | 0.199 | 3*10^5^ |
| T40 | + | 0.1405 | 10^3^ | + | 0.175 | 5*10^7^ |
| T119 | + | 0.124 | 2*10^4^ | + | 0.12 | 10^3^ |
| T69 | + | 0.156 | 4*10^6^ | + | 0.1985 | 10^3^ |
| C14 | + | 0.169 | 10^3^ | + | 0.233 | 3*10^5^ |
| T89 | - |  | 0 | - |  | 0 |
| T6 | + | 0.152 | 10^3^ | + | 0.246 | 10^3^ |
| C13 | + | 0.1505 | 3*10^5^ | + | 0.184 | 10^3^ |
| L39 | + | 0.092 | 10^3^ | + | 0.132 | 3*10^5^ |
| T114 | + | 0.11 | 10^3^ | + | 0.145 | 10^3^ |
| C10 | + | 0.126 | 3*10^5^ | + | 0.145 | 2*10^4^ |
| A31 | + | 0.145 | 10^3^ | + | 0.178 | 10^3^ |
| C42 | + | 0.133 | 10^3^ | + | 0.129 | 2*10^4^ |
| L20 | + | 0.15 | 2*10^4^ | + | 0.1185 | 2*10^4^ |
| T5 | + | 0.127 | 4*10^6^ | + | 0.243 | 10^3^ |
| L8 | + | 0.0915 | 5*10^7^ | + | 0.136 | 2*10^4^ |
| T25 | + | 0.119 | 10^3^ | + | 0.195 | 10^3^ |
| A34 | + | 0.191 | 10^3^ | + | 0.214 | 10^3^ |
| A100 | + | 0.0885 | 10^3^ | + | 0.247 | 4*10^6^ |
| A105 | + | 0.1095 | 2*10^4^ | + | 0.13 | 10^3^ |
| C35 | + | 0.054 | 10^3^ | + | 0.215 | 4*10^6^ |
| C26 | + | 0.196 | 10^3^ | + | 0.1 | 10^3^ |
| A3 | + | 0.146 | 3*10^5^ | + | 0.169 | 10^3^ |
| C119 | + | 0.3445 | 10^3^ | + | 0.555 | 10^3^ |
| T2 | + | 0.085 | 3*10^5^ | + | 0.135 | 2*10^4^ |
| **APEC strain Id** | ***Escherichia*** **phage VaT-2019a** | | | ***Escherichia* phage AG-MK-Basu.2022** | | |
|  | **Spot test** | **Bacterial growth reduction assay (mean decrease of OD 600 nm)** | **Double agar overlay plaque assay (PFU/ml)** | **Spot test** | **Bacterial growth reduction assay (mean decrease of OD 600 nm)** | **Double agar overlay plaque assay (PFU/ml)** |
| C12 | + | 0.062 | 10^3^ | + | 0.247 | 10^3^ |
| A26 | + | 0.0665 | 10^3^ | + | 0.2395 | 10^3^ |
| C90 | + | 0.1954 | 10^3^ | + | 0.164 | 10^3^ |
| C45 | + | 0.062 | 10^3^ | + | 0.125 | 2*10^4^ |
| C53 | + | 0.063 | 3*10^5^ | + | 0.145 | 3*10^5^ |
| A14 | + | 0.085 | 5*10^7^ | + | 0.178 | 10^3^ |
| A13 | - |  | 0 | + | 0.123 | 10^3^ |
| T110 | + | 0.1375 | 10^3^ | + | 0.263 | 10^3^ |
| A16 | + | 0.065 | 10^3^ | + | 0.264 | 4*10^6^ |
| A107 | + | 0.0747 | 4*10^6^ | + | 0.185 | 4*10^6^ |
| A38 | + | 0.057 | 4*10^6^ | + | 0.1125 | 5*10^7^ |
| L83 | + | 0.071 | 5*10^7^ | + | 0.1795 | 10^3^ |
| C1 | + | 0.0715 | 2*10^4^ | + | 0.1545 | 2*10^4^ |
| A6 | + | 0.1135 | 10^3^ | + | 0.1085 | 10^3^ |
| T38 | + | 0.0755 | 2*10^4^ | + | 0.21 | 5*10^7^ |
| A42 | + | 0.0755 | 10^3^ | + | 0.195 | 4*10^6^ |
| A27 | + | 0.065 | 10^3^ | + | 0.12 | 10^3^ |
